# An extension of the BioAssay Ontology to include pharmacokinetic/pharmacodynamic terminology for the enrichment of scientific workflows

**DOI:** 10.1186/s13326-023-00288-6

**Published:** 2023-08-11

**Authors:** Steve Penn, Jane Lomax, Anneli Karlsson, Vincent Antonucci, Carl-Dieter Zachmann, Samantha Kanza, Stephan Schurer, John Turner

**Affiliations:** 1 Pfizer Inc, 1 Portland Street, Cambridge, MA 02139 USA; 2 Scibite an Elsevier Company, Scibite Ltd, Biodata Innovation Centre, Wellcome Genome Campus, Hinxton, Cambridge, CB10 1DR UK; 3 Merck & Co Inc, 126 E Lincoln Ave, Rahway, NJ 07065 USA; 4 Sanofi-Aventis Deutschland GmbH, R&D / Integrated Drug Discovery, Industriepark Hoechst, Frankfurt am Main, H831 C.0156, 65926 Germany; 5 Department of Chemistry, University of Southampton, Highfield Campus, University Road, Southampton, SO17 1BJ UK; 6 Department of Cellular and Molecular Pharmacology, Miller School of Medicine, University of Miami, Miami, FL 33136 USA

**Keywords:** ADME (Absorption, Distribution, Metabolism, Excretion), BioAssay, Drug safety, Electronic lab notebook, FAIR (Findable, Accessible, Interoperable and Reusable) principles, Ontologies, Pharmacology, Pharmacokinetics, Pharmacodynamics, Semantic, Unstructured text

## Abstract

With the capacity to produce and record data electronically, Scientific research and the data associated with it have grown at an unprecedented rate. However, despite a decent amount of data now existing in an electronic form, it is still common for scientific research to be recorded in an unstructured text format with inconsistent context (vocabularies) which vastly reduces the potential for direct intelligent analysis. Research has demonstrated that the use of semantic technologies such as ontologies to structure and enrich scientific data can greatly improve this potential. However, whilst there are many ontologies that can be used for this purpose, there is still a vast quantity of scientific terminology that does not have adequate semantic representation. A key area for expansion identified by the authors was the pharmacokinetic/pharmacodynamic (PK/PD) domain due to its high usage across many areas of Pharma. As such we have produced a set of these terms and other bioassay related terms to be incorporated into the BioAssay Ontology (BAO), which was identified as the most relevant ontology for this work. A number of use cases developed by experts in the field were used to demonstrate how these new ontology terms can be used, and to set the scene for the continuation of this work with a look to expanding this work out into further relevant domains. The work done in this paper was part of Phase 1 of the SEED project (Semantically Enriching electronic laboratory notebook (eLN) Data).

## Background

Deloitte’s 2019 Global Life Sciences Outlook^1^ identifies strategic transformation as the core of future business models for life science companies, with broadly shared data acting as the currency to ensure real value is delivered to patients [1]. However, the volume of data (documents, experimental data etc) produced by scientific research is growing at an exponential rate, and researchers are struggling to effectively manage, curate and extract the required knowledge from these materials [2]. Part of this challenge results from the amount of data that is recorded as unstructured text in laboratories, without a concerted effort to use standardized terminologies.

FAIR data are data which meet principles of Findability, Accessibility and Interoperability, with the ultimate aim of making that data reusable [3]. Unstructured data poses several key issues to making scientific research FAIR. The data is not interoperable or particularly re-usable in its current form and cannot programmatically be linked with other relevant data, rendering the level of insight that can be gained from it to be superficial [4]. Further, the lack of consistent metadata makes the data both difficult to find and inaccessible [5].

A potential solution to this is to semantically enrich unstructured text with consistent scientific terminologies, both within and across scientific domains. Ontologies are semantic models of a specific domain, built by humans and readable by machines, and provide rich descriptions of things (classes) and how they relate to each other. The life-sciences domain is particularly well-served for ontologies where they have been widely used and developed for the last 20 years [6]. They provide a mechanism to unambiguously tag concepts found within data, thus expanding the range of potential insights possible from scientific research. Such a capability would be very useful in ELNs to enable scientists to link together relevant documents and gain new insights from others work.

ELNs were initially developed as a means to transition from paper-based record keeping to electronic record keeping in the laboratory as a means to provide intellectual property protection, and simultaneously provided a platform for laboratory data management and workflow execution. ELNs have evolved significantly over the past two decades in terms of their technology platforms (including cloud-hosted products) and embedded data analysis capabilities, which has dramatically improved the user experience, extensibility, performance, and total cost of ownership to an organization. However, limited progress has been made to date to adopt consistent ontologies or data models in ELN products to enable simpler data sharing and data consumption across scientific domains and software applications.

This was the driving motivation behind the proposal and subsequent initiation of the SEED project [semantic enrichment of Electronic Laboratory Notebook (ELN) data] in the Pistoia Alliance^2^ [7]. Pfizer^3^ [8] recommended the project to Pistoia as a means to create a set of ontologies for contextualizing ELN data across Pharma and the life sciences. This paper details the work done on Phase 1 of this project, including narrowing down the scope of the first scientific domain of interest to PK/PD, identifying the relevant ontologies for use and adaption, and ultimately the work that was done to produce a new set of ontology terms for this area. The SEED project includes extensive representation across the Pharma industry, ELN Providers, and Semantic providers who are co-authors on this manuscript. Specifically, members aforementioned, identified the value of PK/PD domain in regards it interoperability across the Pharmaceutical industry and it necessity in support of the drug development lifecycle, but most importantly the gaps in an overarching industry assay ontology that fully supports this domain.

## Construction and content

We performed an initial review of publicly available ontologies to understand which provided coverage of the PK/PD assay domain. The public ontologies that were included in the reviewed were: BioAssay Ontology (BAO) v2.6 [9], National Cancer Institute Thesaurus (NCIt) [10] (v20.06e), Experimental Factor Ontology (EFO) [11], Chemical Methods Ontology (CHMO) [12], and the Ontology of Biomedical Investigations (OBI) [13].

**Fig. 1:**
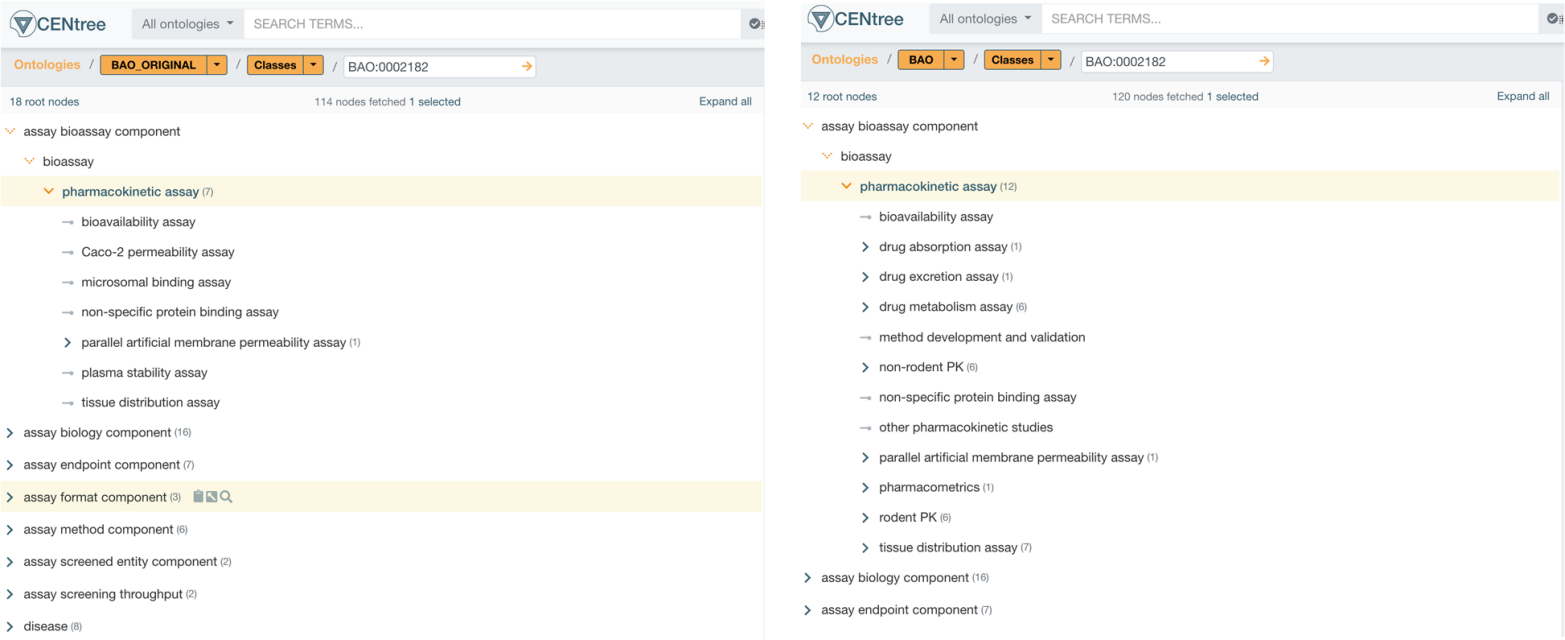
A CENtree screenshot of BAO before and after the addition of new PK assay classes (Jupp, et al. Centree: An ontology management and editing platform for the life-sciences, unpublished)

We found that BAO contained the most relevant terms for PK/PD and some pre-existing ontology structure related to these areas. For example, the parent term “pharmacokinetic assay” and some subclasses were already part of BAO. In addition, BAO also contained other relevant metadata types such as endpoints, assay types and detection methods (see Fig. 1). We therefore decided to use BAO as the base ontology and purpose an expansion of the ontology to include PK/PD assay classes. Several relevant classes were also found in the NCIt and adhering to the principle of ontology re-use wherever possible, we decided that these classes should be imported directly into BAO rather than creating new classes. The review entailed a qualitative evaluation between the public ontologies and extracted Pharma PK/PD assay data from internal LIMS and Assay registration applications supplied by SME’s across 3 pharma. This review was executed by Pharma PK/PD and ontology SMEs. Expert SME and ontology input was utilized to align, review and agree ADME assay Ontology coverage and alignment.

A list of commonly used PK/PD assays and assay related terms were proposed by project members representing the pharmaceutical industry. Additional information for developing the new classes was gained from reviewing various sources including industry guidance documents published by the International Council for Harmonisation of Technical Requirements for Pharmaceuticals for Human Use (ICH) International Council for Harmonisation of Technical Requirements for Pharmaceuticals for Human Use (ICH) [14] and FDA [15], NIH [16],scientific publications [17–20] and Wikipedia. The collated terms and new classes were integrated into the BAO ontology structure using the ontology management tool CENtree^4^ and appropriate parent classes and synonyms added. The final version of the new classes and synonyms was reviewed by project members and subject matter experts (SMEs) who provided additional suggestions and edits. This feedback was incorporated, and a final list of 198 new classes and synonyms were submitted and subsequently added to BAO.

In addition to novel terms, 21 classes from NCI thesaurus were imported and integrated into BAO [10]. Requested new classes or any modifications of current classes were submitted in the form of a comma separated value spreadsheet using a simple template. This bulk submission spreadsheet was shared with the BAO curation team to review the requests. The changes were brought up at the next curation meeting for BAO where domain experts were consulted to ensure that none of the suggested changes conflict with BAO or current knowledge on the subjects. BAO curator feedback was shared with the SEED team for correction or clarification. As an example, one of the discussion points was changing the hierarchy of the term non-specific protein binding assay (BAO:0002531) from sub-class of pharmacokinetic assay (BAO:0002182) to the subclass tissue distribution assay (BAO:0002532). However, after consideration with the curation team, it was decided to keep the original classification and the SEED team agreed to BAO curators’ reasoning. The SEED team also noticed that many of the terms under the assay methods were ambiguous and could be seen as methods or assays and has started an internal review of these terms.

BAO’s modular and hierarchical architecture of internal and imported vocabulary and axiom modules facilitates maintenance and updates [21]. In particular, updates to vocabularies and simple axioms, such as in this project, can relatively seamlessly be incorporated. Updates are now further supported by an in-house tool called OntoJog^5^ that automates the construction of the BAO ontology in OWL-DL from the BAODB, a PostgreSQL-based database that contains vocabularies and basic axioms, and more complex “template” axiom files (publication in preparation).

Following standard ontology development methodology for the SEED project, the largely manual knowledge acquisition and formalization process by the SEED and BAO teams resulted in a structured document that included new BAO terms with labels, synonyms and their parent superclasses, and modifications to existing BAO terms, including additional synonyms and changes in class hierarchy. The next step was to incorporate these changes into the BAODB. For quality control, the updates to BAODB were first simulated via an in-memory only “dry run” assigning temporary IDs to new classes while referencing the database to check for missing fields, malformed requests and overall integrity issues that might require manual intervention. Following that QC and validation step, BAODB was updated including permanent IDs / URIs. A provisional version of BAO that included all changes was then created using OntoJog. This BAO release candidate was then distributed to the curation team for review and manual QC of possible issues or typos introduced during the generation of the new version. In addition, a change report was produced to allow review or all URI-, class labels- and -hierarchy changes compared to the previous release. Once all issues were resolved a new version of BAO was released via the BAO website from where it was picked by the BioPortal.

**Fig. 2:**
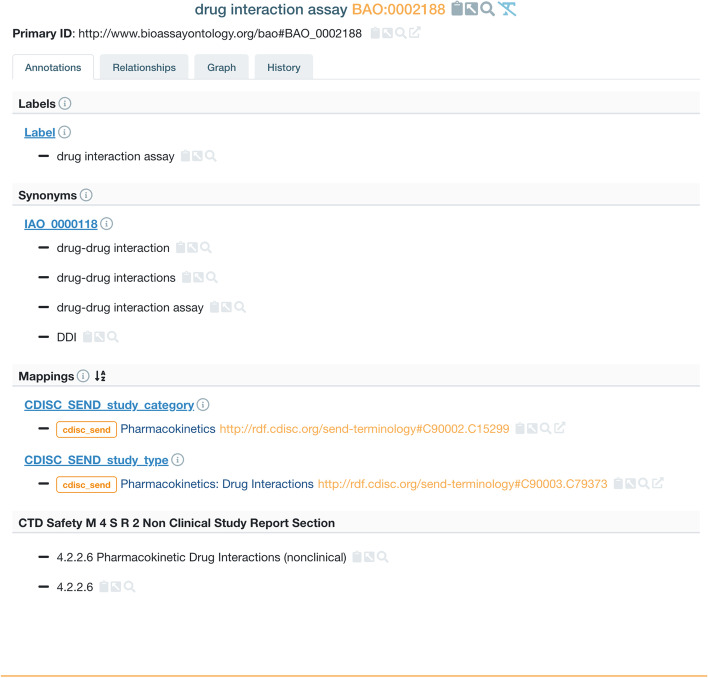
Addition of synonyms and mappings (Jupp, et al. Centree: An ontology management and editing platform for the life-sciences, unpublished)

The main area of BAO that was expanded was under the existing BAO term ‘pharmacokinetic assay’ (http://www.bioassayontology.org/bao#BAO_0002182). The key assay classes added were drug absorption, drug excretion, drug metabolism and subclasses under existing class “tissue distribution assay” (http://www.bioassayontology.org/bao#BAO_0002532). When adding classes and individual terms and assays, a key consideration was the possibility to map to standards such as CDISC SEND (Standard for Exchange of Nonclinical Data) [22] to support submissions to regulatory authorities and to annotate assay terms with corresponding sections of for example M4S Nonclinical Study submission reports (see Fig. 2).

Many scientific terms have multiple commonly used names and acronyms and scientists often use different terms interchangeably to describe the same thing. For example, drug interaction study can be annotated as DDI study, drug interaction assay, drug-drug interaction or just DDI. To cover the variation in language, we also added more than 700 synonyms to both new and existing classes and terms. (see Fig. 2).

**Fig. 3:**
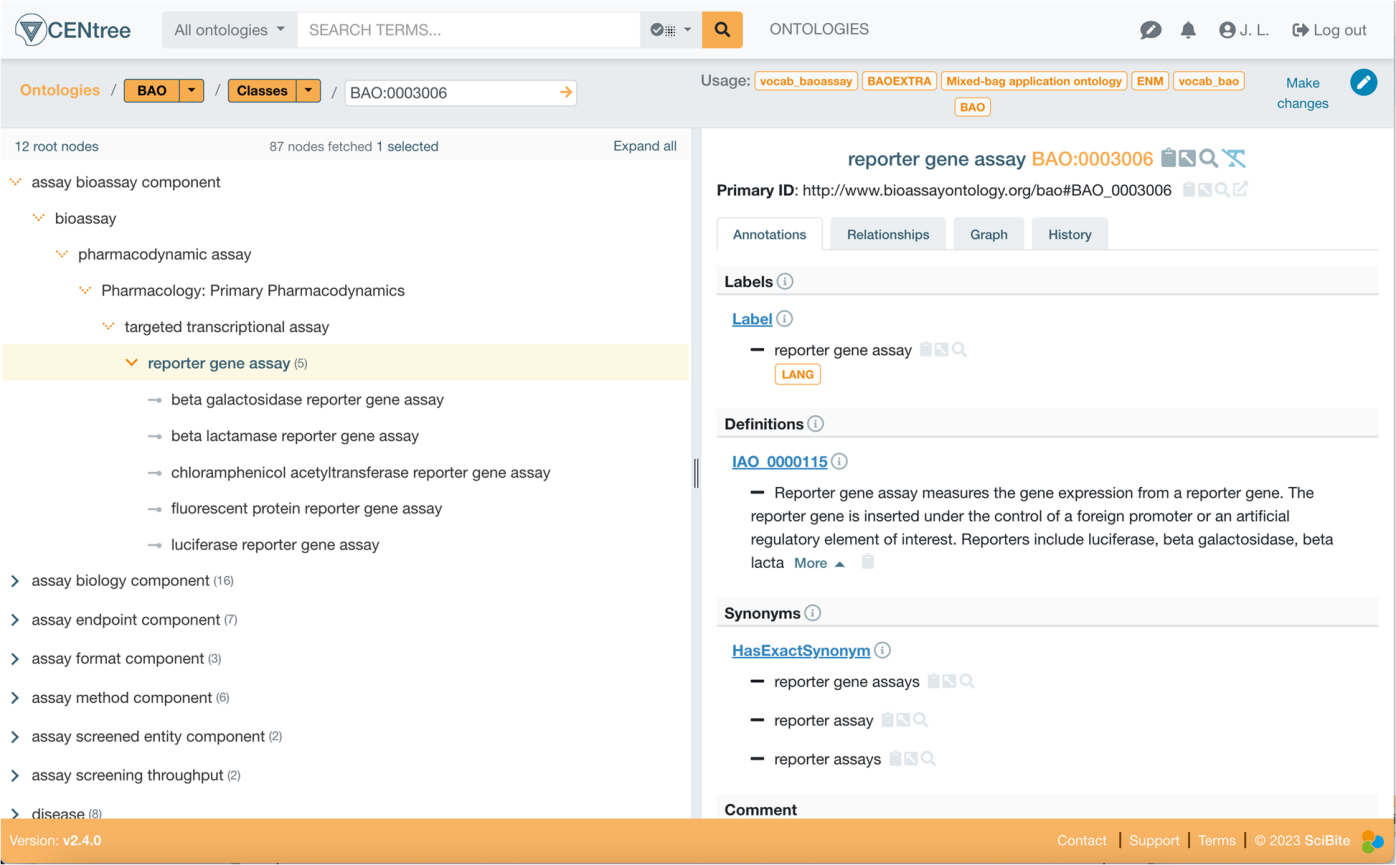
Synonyms and some subclasses were added to the pharmacodynamic domain (Jupp, et al. Centree: An ontology management and editing platform for the life-sciences, unpublished)

The other main area enriched was Pharmacodynamic (PD), under the BAO term “pharmacodynamic assay”. This domain is already well covered in BAO, and so the main work here was to add synonyms and import some high-level terms from NCIt such as “Pharmacology:Primary Pharmacodynamics” to help with the overall structure of the ontology (Fig. 3).

## Utility and discussion

### General problem description

There are no known public sources for CDISC Standard for Exchange of Nonclinical Data (SEND) data, so organizations will likely need to build internal databases or external sharing arrangements with CRO’s to store data for local operations [23].

**Fig. 4:**
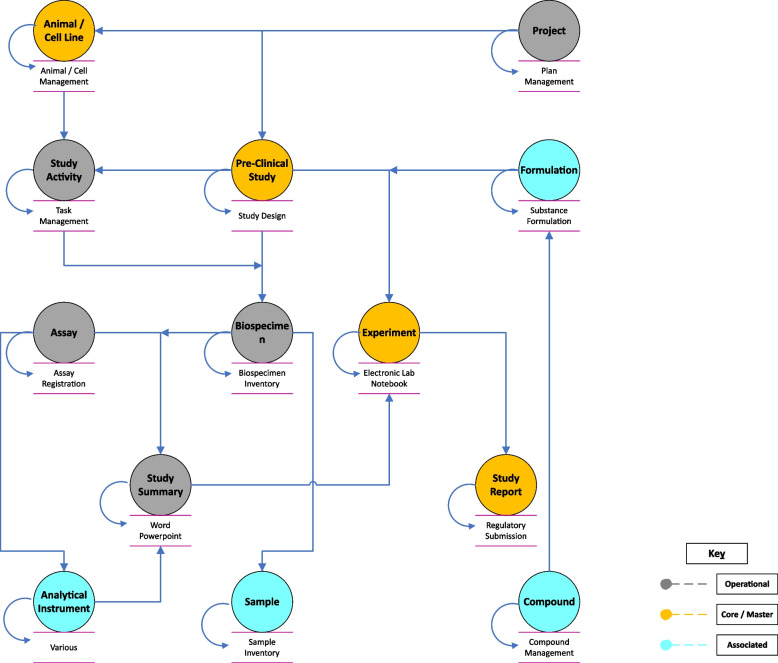
Representative Model of a Pre-Clinical eco-system

Due to a lack of unambiguous standardization within and across systems used in the Pre-Clinical eco-system as illustrated in the representative example in Fig. 4, data integration historically has been and often remains a manual task [24]. Data processing is often executed iteratively whereas data is generated continuously at varying scales of biological complexity. Moreover, Pre-Clinical data is often stored in an unstructured manner in siloed systems that are not properly connected to the rest of the eco-system [25].

Assay data classification based on PK and PD classes incorporated into BAO will help to standardize Pre-Clinical (In vivo, Ex vivo, and In vitro) internal and external data sources according to the FAIR principles (Findability, Accessibility, Interoperability, and Reusability) [3]. This approach explores a framework for development of end point specific results (e.g., histopathology findings, assay measurements) assembled in a user-defined subset of studies for cross study analysis.

If all Pre-Clinical data can be stored consistently using semantically rich domain-based ontologies along with contextual metadata as a data set, seamless flow of data across the integrated eco-system will provide an opportunity to apply large scale data analytic approaches [26, 27] for cross study analysis in a cohesive manner.

In the following paragraphs four practical Use Cases are described that showcase the usefulness of standardized assay metadata.

### ADME and PD assay Registration with metadata assignment based on standard bioassay ontology BAO (SANOFI)

**Fig. 5:**
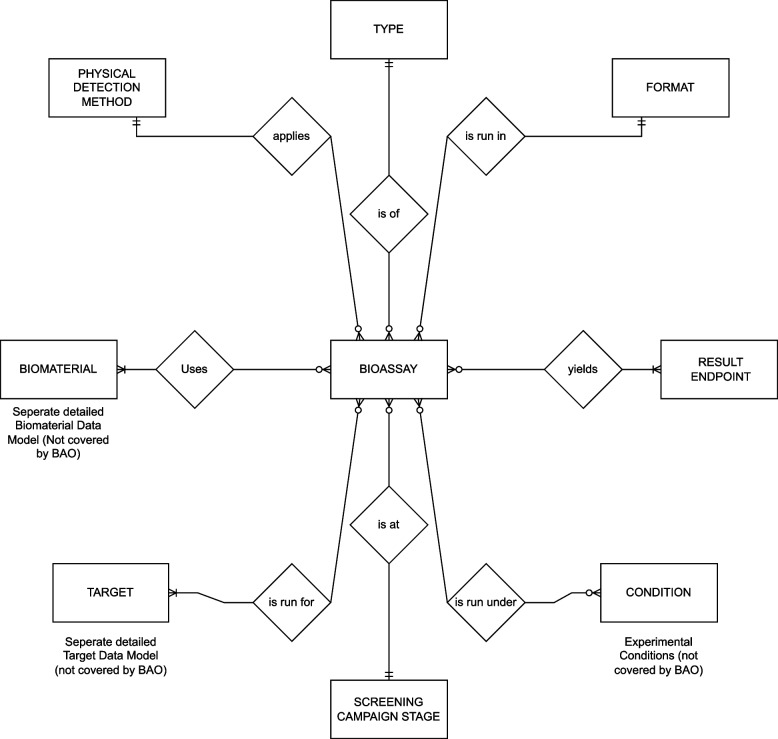
Sanofi’s Assay Data Model

Sanofi’s assay data model was redesigned based on the new ADME and PD standard assay ontology, which was defined and integrated into BAO by the Pistoia SEED Project Team. Now all ADME and PD assay metadata are standardized and aligned with a minimum set of ontology classes from BAO (Fig. 5).

It is planned to use SciBite’s CENtree Ontology Management Platform [28] to feed BAO classes via API into an application for assay registration. The objective is to register all assays with a standardized set of mandatory metadata according to the FAIR principles; particularly this will improve

- **Findability** to support Project progression
- **Interoperability** with internal and external data (coming for instance from a CRO)
- **Reusability** for data analytics and data science applications.

### Semantic tagging of unstructured lab reports based on BAO to identify and extract standardized metadata for assay registration in central repository (SANOFI)

**Fig. 6:**
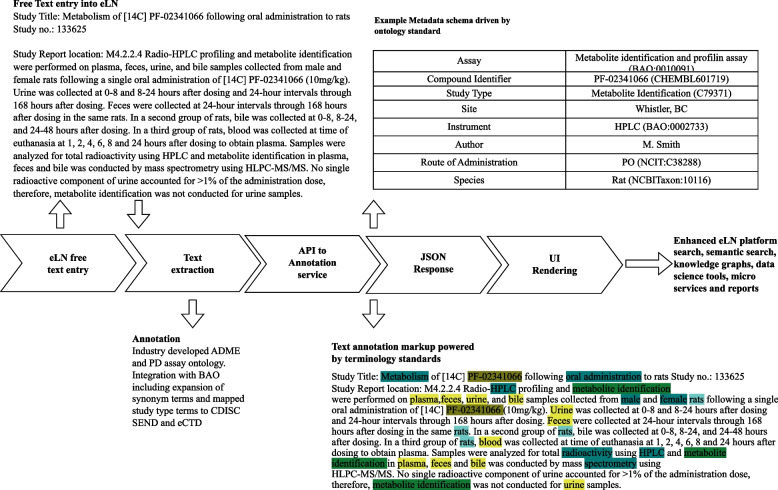
SEED eLN unstructured data annotation workflow

Ontologies managed in CENtree are planned to be used as source vocabularies in SciBite’s TERMite Text Analytics and Semantic Enrichment platform [29] that can, in turn, be used for automatic annotation of unstructured data with the same ontologies used for structured data. For example, a Lab Report describing an ADME assay will be uploaded into an eLN with embedded semantic enrichment function and automatically annotated with the ADME/PD classes from BAO. The annotation will be used to automatically assign BAO classes as metadata to the assay. The ultimate goal is to extract as many metadata as possible from unstructured reports which will be facilitated by adding reasonable synonyms to the BAO classes in in CENtree and TERMite (Fig. 6).

### Semantic tagging of unstructured content to enable identification and selection of data for submission reports for module 4 of an eCTD (Pfizer)

Another Use Case relying on BAO classes is pursued by Pfizer to support regulatory study report creation. More specifically, the goal is to combine the use cases above, semantically enabling both structured and unstructured data. Tagging unstructured content in the eLN, and application of the consistent ontology standards from the assay registration system into the eLN to benefit identification and selection of data for ADME and PD submission reports supporting Module 4 of an eCTD. ADME and PD experiments and studies available in an eLN or LIMs will be semantically tagged with the BAO class “bioassay type ADME” or “bioassay type PD” in addition to the assay term name and any mapping to CDSIC SEND Study type and/or CTD Safety M4S(R2) Non-clinical study report section (to be added to BAO). This will enable users in part (with addition of data specifying Compound, Species, Route of Admin etc) to search for the specific experiment data using the newfound FAIR data infrastructure to drive advances in automated regulatory study report creation.

### Modular design of data capture in the electronic notebook using ontologic terminology to facilitate downstream data consumption (Merck)

An additional use case for the subject ontology of this manuscript, as well as other well-formed public ontologies, is to drive the creation of modular and consistent data capture at the point of data creation in the electronic notebook. Modular electronic notebooks typically have an overarching information model that organizes high level experiment concepts in a single meta model. However, often these tools allow individual users / organizations to configure the electronic notebook via templates to consistently structure and contextualize selected content in experiments. There are many scientific workflows across the various scientific domains addressed with an electronic notebook, so driving consistency in data capture to yield the downstream benefits during data consumption (data aggregation, analytics, visualization, data science, etc) can seem daunting. However, if approached in a modular fashion with reusable content across templates where possible, one creates manageable-sized data standardization problems to solve.

To drive standardized data capture in the electronic notebook, Merck is decomposing typical experiment designs across various scientific domains into the frequently reused smaller components that are either generally used across experiments, or typically used within a specific scientific domain. A centralized master conceptual model organizes ontologies across scientific domains into a single context and source of truth across domains to ensure consistent use of the concept. Finally, these ontologies are embedded in the reusable elements in the notebook, which are used to compose the experimental templates used for standardized data capture. The end result should be a better user experience for scientists who can quickly capture data in a more consistent fashion, knowing it will simplify on-demand downstream data mining and analysis for themselves and their colleagues. Product owners / administrators will find the semantically enabled electronic notebook more scalable since content is intentionally reused across templates to drive standardization. Holistically, embedding these ontological terms into electronic notebook data capture links each experiment back to the master conceptual data model, creating the possibility of greater data exploration across not only the notebook but other knowledge assets leveraging the ontologies as well.

## Conclusions

Data standardization efforts by their very nature should be collaborative efforts that bring various subject matter experts together across scientific, information technology, and semantics domains to ensure diverse perspectives are incorporated in the work product. Specifically, this is an area where pre-competitive collaboration is absolutely essential to ensure the output is likely to be judged as broadly useful and widely-accepted. The electronic notebook platform is at the heart of research intensive organizations, and the data the flows through it gives life to the organization both today and tomorrow. Therefore, it’s the ideal place to invest in semantic enrichment at the point of original data capture (the source) prior to broad data distribution of the data throughout the organization. This ensures that there’s a consistent and reusable context and structure present in the data than can always be leveraged, and where needed further extended. The authors encourage other subject matter experts to participate in pre-competitive standardization efforts to ensure domains of importance are addressed in a timely fashion and then published in an appropriate public ontology for all to use.

## What’s next

Extension of the Ontology work beyond PK and PD domains to encapsulate Drug Safety assays is underway. Collection of terms for Drug safety assay/study types to be integrated into Bioassay ontology is agreed upon and planned. This will extend coverage to all the Nonclinical studies in Module 4 of an eCTD (electronic Common Technical Document) in support of an NDA (New Drug Application) submission. This will allow us to extend the use-cases presented earlier from Sanofi, Merck, Astra Zeneca, and Pfizer into the Drug Safety domain.

Building on the foundation for text annotation using the ontology development work for assays, the SEED Project team is gathering assay relationships (mapping) and annotations to enhance PK and PD assay ontology (in addition to the Drug safety assay ontology when this output is available). Adding additional data in the form of attributes, mappings and annotations creates relationships between ontology classes and helps to describe and define them. The relationships formed between objects within an ontology and to other ontologies and/or standards form a framework for the creation of a graph ontology/knowledge graph.

Work continues with the many eLN providers that are valuable members of the SEED team to incorporate the enhanced ontologies to their platforms. And with linkage to a knowledge graph the ontologies and mapped relationships connects the electronic lab-notebook capture for each experiment back to a conceptual model. This can facilitate a holistic exploration of data across domains and equally across applications utilizing the conceptual model framework, uncovering previously hidden insights.

Longer term, public ontologies from other areas of Pharmaceutical development should be evaluated for completeness and extended where necessary to document preferred terminology and relationship mapping of importance to consistently contextualize ELN data Possible examples include analytical methods development, formulations sciences, and bioprocess development just to name a few.

## Data Availability

The datasets generated and/or analysed during the current study are available in the BioAssay Ontology repository, (BAO). The most current SEED implementation of BAO is version 2.7.3 available on the BioPortal https://bioportal.bioontology.org/ontologies/BAO and on GitHub: https://github.com/BioAssayOntology/BAO/releases/tag/v2.7.3.. The most current version of BAO is available at http://bioassayontology.org/bao/bao_complete.owl.

## Abbreviations

ADME: Absorption, Distribution, Metabolism and Excretion
BAO: Bioassay Ontology
BAODB: Bioassay Ontology Database
CDISC SEND: Clinical Data Interchange standards Consortium - Standard for Exchange of Nonclinical Data
CRO: Contract Research Organization
DDI: Drug Drug Interaction
CHMO: Chemical Methods Ontology
eCTD: Electronic Common Technical Document
EFO: Experimental Factor Ontology
ELN: Electronic Laboratory Notebook
NCIt: National Cancer Institute Thesaurus
NDA: New Drug Application
OBI: Ontology of Biomedical Investigations
PD: Pharmacodynamics
PK: Pharmacokinetic
PK/PD: Pharmacokinetic/Pharmacodynamic
SEED: Semantic Enriching ELN Data

## Footnotes

1 Deloitte’s 2019 Global Life Sciences Outlook is Deloitte’s annual (2019) viewpoint on trends and areas of focus in the Life sciences industry

2 The Pistoia Alliance is a not-for-profit organization with over 200 member companies across the life science ecosystem collaborating across more than 25 projects and communities to advance science.

3 Pfizer is a global leading research-based biopharmaceutical company

4 CENtree is an ontology editing and management platform, available under commercial licence which was waived for this project (Jupp, et al. Centree: An ontology management and editing platform for the life-sciences, unpublished). It was chosen for this project because of its simple user interface and its support of multiple-user editing and governance. In addition, members of the project team were already skilled in using the tool., developed by SciBite (Jupp, et al. Centree: An ontology management and editing platform for the life-sciences, unpublished)

5 OntoJog aids in the generation of OWL files from the BAO relational database. It is available on GitHub at https://github.com/schurerlab/OntoJog.
